# Hospital Administration in War-Time

**Published:** 1915-12-25

**Authors:** 


### The Hospital

The Workers' Newspaper of Administrative Medicine and Institutional Life, Administration, National Insurance and Health.

No. 1541, Vol. LIX. SATURDAY, DECEMBER 25, 1915. PRICE ONE PENNY.

### HOSPITAL ADMINISTRATION IN WAR-TIME.

The administration of a hospital is a task which includes many different interests, and to keep these in due proportion needs much attention, intelligence, good judgment, and tact. In the very nature of things there easily arise competition and conflict in regions where co-operation and union are conditions essential to full success. Apart from less broad points of view, there is on one side the economic and philanthropic aspect of the institution, and on the other its professional or scientific relations. The former necessarily appeals especially to the lay manager and the latter to the medical staff. It is hardly too much to say that the supreme duty of all concerned is to secure the proper adjustment and harmony of these two motives in the general scheme of administration. When this end is attained, not only is the domestic atmosphere of the hospital undisturbed, but its claim for public confidence and support comes to full vigour. The general recognition of this truth finds expression in the common practice of associating with laymen on the governing body a certain number of representatives of the medical staff. Meeting in a common council the various interests have thus an opportunity for complete and accurate statement, and one of the principal responsibilities of the chairman and his colleagues is to unite them in such a fashion as shall promote the maximum usefulness of the hospital. There are occasions when such an end is not easy to achieve, and the explanation of the difficulties is found sometimes in persons and sometimes in events. At the present moment there are circumstances tending to sharpen differences which are in greater or less degree inherent in the position, and these call, therefore, for a special measure of forbearance and for an added grace of temper. Almost everywhere the medical staff is much over-worked, and committee service and the details of administration become in such circumstances peculiarly trying and irksome. The absence of colleagues means larger duties for those who remain, and out of this grows strain of both time and endurance. On the other hand, it is well for the doctors to remember that the new burdens of the day do not fall only on their shoulders, and that laymen also feel the tension and the anxieties of the hour. Thus the appeal comes to both sides to cultivate consideration and forbearance, and to strive to appreciate interests and concerns other than those which make an immediate and individual claim.

One danger of the present situation is that the medical staff under the pressure of events detaches itself from the problems of general administration. Their representatives on the governing body ought never to be regarded, nor should they regard themselves, as merely concerned with interests which are solely or narrowly professional. Equally with their colleagues they have the duty and opportunity of contributing to the efficiency and smooth working of all the departments of hospital life and activity. Indeed, no self-respecting man ought to consent to sit on an administrative council except under the condition just mentioned. To isolate one group of members as having no responsibilities or voice except when questions of medical policy are in debate is an invidious and wholly inexcusable procedure, and a council which encourages or permits it is steering towards disaster. Inevitably in such circumstances any differences which may arise between the lay and the medical points of view will be aggravated into hostility and conflict, and no greater misfortune can well befall the conduct of a hospital. Hence, troublesome and difficult though it be in these times of pressure, medical representatives must be urged to keep in active touch with all the life of the hospital as this is presented for survey and judgment at the meetings of the authoritative council. And laymen whose occupations allow a more confident adjustment of their time must be prepared to make allowances for some degree of unpunctuality and irregularity of attendance. Another point which may be urged is that in the conduct of committee business there should be the cultivation of dispatch. Such a course may be commended at all times, but it is especially urgent in circumstances which multiply the claims made on the time and strength of those engaged in medical practice. In this respect, as all the world knows, much depends on the efficiency of the chairman, and happy, particularly just now, is the committee which has at its head a competent man of affairs. It is often said that doctors are not good men of business, but even if we allow this for the sake of argument, it is certain that they are very ready to appreciate the display of this virtue in others. The hospital chairman who by tactful expedition saves the time of his committees will at the present juncture receive a special measure of gratitude from his medical colleagues.

Another result which may readily follow from non-attendance of the medical members of hospital governing councils is some failure on the part of the staff to realise the position of the lay directors in relation to the public. Take, for example, the question of the maintenance or of the suspension of the facilities offered by a hospital for the treatment of patients. It is easy, and not unnatural, for the staff to meet together and to recommend the directors to cut down these facilities on the ground that the acting members of the staff are seriously reduced in number. But the directors must needs be reluctant to take such a step unless the necessity can be justified beyond challenge. They have to consider the opinion of subscribers and still more they have to bear in mind the claims of the public. A hospital is not a private venture which can be opened or closed at will. It has public responsibilities which must not be yielded save under absolute compulsion. It lies with the medical staff to appreciate this position and to co-operate with the lay directors in securing its satisfaction.

